# Both ciliary and non-ciliary functions of Foxj1a confer Wnt/β-catenin signaling in zebrafish left-right patterning

**DOI:** 10.1242/bio.012088

**Published:** 2015-10-02

**Authors:** Ping Zhu, Xiaolei Xu, Xueying Lin

**Affiliations:** Department of Biochemistry and Molecular Biology, Mayo Clinic, Rochester, MN 55905, USA

**Keywords:** *Charon*, Ciliogenesis, Foxj1a, Wnt/β-catenin signaling

## Abstract

The Wnt/β-catenin pathway is implicated in left-right (LR) axis determination; however, the underlying mechanism remains elusive. Prompted by our recent discovery that Wnt signaling regulates ciliogenesis in the zebrafish Kupffer's vesicle (KV) via Foxj1a, a ciliogenic transcription factor, we decided to elucidate functions of Foxj1a in Wnt-regulated LR pattern formation. We showed that targeted injection of *wnt8a* mRNA into a single cell at the 128-cell stage is sufficient to induce ectopic *foxj1a* expression and ectopic cilia. By interrogating the transcription circuit of *foxj1a* regulation, we found that both Lef1 and Tcf7 bind to a consensus element in the *foxj1a* promoter region. Depletion of Lef1 and Tcf7 inhibits *foxj1a* transcription in the dorsal forerunner cells, downregulates cilia length and number in KV, and randomizes LR asymmetry. Targeted overexpression of a constitutively active form of Lef1 also induced an ectopic protrusion that contains ectopic transcripts for *sox17*, *foxj1a*, and *charon*, and ectopic monocilia. Further genetic studies using this ectopic expression platform revealed two distinct functions of Foxj1a; mediating Wnt-governed monocilia length elongation as well as *charon* transcription. The novel Foxj1a-*charon* regulation is conserved in KV, and importantly, it is independent of the canonical role of Foxj1a in the biosynthesis of motile cilia. Together with the known function of motile cilia movement in generating asymmetric expression of *charon*, our data put forward a hypothesis that Foxj1a confers both ciliary and non-ciliary functions of Wnt signaling, which converge on *charon* to regulate LR pattern formation.

## Abstract (precis)

**Summary:** Using a targeted overexpression platform, we showed that Wnt activation induces ectopic *foxj1a* expression and ectopic cilia formation, and revealed two distinct roles of Foxj1a in conferring Wnt-governed left-right patterning.

## INTRODUCTION

Vertebrates display a distinct left-right (LR) asymmetry in the disposition of their internal organs (Raya and Belmonte, 2006). The specification of LR axis can be divided into at least two stages: (1) the generation of asymmetric signals around the mouse embryonic node or its derivatives, such as the zebrafish Kupffer's vesicle (KV), and (2) transferring of the asymmetric cues to the lateral plate mesoderm (LPM) and internal organs (Nonaka et al., 2002; Essner et al., 2005; Kramer-Zucker et al., 2005; Hirokawa et al., 2006). Motile cilia have been shown to play a crucial role in establishing LR asymmetry at the first stage. Motile cilia occur as a single cilium extending from cells in the node or KV, where coordinated cilia beating generates a leftward fluid flow that is essential for generating asymmetric signals around the node, such as left-side downregulation of Cerberus/Dan family members *Coco* (*Xenopus*), *Cerl2* (mouse), and *charon* (medaka and zebrafish) (Hojo et al., 2007; Oki et al., 2009; Schneider et al., 2010; Schweickert et al., 2010; Nakamura et al., 2012). The asymmetric expression of these Nodal antagonists promotes Nodal (Spaw in zebrafish) activity on the left side of the node, which is then transferred and propagated to the left LPM (Kawasumi et al., 2011).

The Wnt/β-catenin pathway has been shown to play a role in regulating LR pattern formation. Wnt activation by KV-specific overexpression of stabilized β-catenin or KV-specific depletion of Axin, a Wnt/β-catenin antagonist, results in randomized side-specific gene expression (Schneider et al., 2008), whereas global Wnt activation at levels not causing severe embryo malformation affects the competence of heart field and gives rise to no-looping heart without appreciably altering asymmetric gene expression in LPM (Carl et al., 2007; Lin and Xu, 2009). In contrast, loss of function of Wnt leads to randomized side-specific gene expression and randomized organ laterality as noted in mouse *Wnt3a* mutant, as well as zebrafish *wnt3a*, *wnt8a*, *β-catenin* and *fzd10* morphants (Nakaya et al., 2005; Lin and Xu, 2009; Caron et al., 2012; Zhang et al., 2012).

At the zebrafish LR organ KV, we and others showed that inhibition of Wnt signaling results in shorter and fewer cilia, disordered fluid flow, downregulation of *charon*, and reduced cell proliferation (Lin and Xu, 2009; Caron et al., 2012; Zhang et al., 2012). Further investigation suggests that Wnt/β-catenin signaling regulates ciliogenesis via transcriptional control of *foxj1a* (Caron et al., 2012), a forkhead domain-containing transcription factor that is necessary for ciliogenesis in multiciliated cells of the mouse airway epithelial cells and monocilia biosynthesis in the zebrafish KV and *Xenopus* gastrocoel roof plate (GRP, frog equivalent of mouse node) (Chen et al., 1998; Brody et al., 2000; Stubbs et al., 2008; Yu et al., 2008). Consistent with Wnt-*foxj1* regulation, a recent study in *Xenopus* reported expansion of *foxj1* expression domain in the GRP by ectopic expression of β-catenin (Walentek et al., 2012). However, the Wnt-Foxj1-ciliogenesis-LR asymmetry hypothesis is not completely compatible with observations in the mouse. It has been shown that Wnt3a deficiency is associated with lack of coexpression of mechanosensing proteins PC1 and PC2 in the cilium without affecting cilium structure and motility in the node (Nakaya et al., 2005). While Foxj1 is expressed in the mouse node and deletion of the gene results in randomized LR asymmetry as Foxj1a does in zebrafish, nodal cilia are present in the Foxj1 knockout mice (Chen et al., 1998; Brody et al., 2000; Stubbs et al., 2008; Yu et al., 2008). Together, these inconsistencies suggest other, unrecognized functions of Foxj1 in LR pattern formation, prompting the present study to further interrogate functions of the Wnt-Foxj1 signaling axis in LR patterning.

Here, we present biochemical and genetic evidence to indicate that Wnt signaling directly regulates *foxj1a* transcription in KV through cooperative action of Lef1 and Tcf7. Using a targeted overexpression platform, i.e. injection of mRNAs into a single cell at the 128-cell stage (Agathon et al., 2003), we showed that Wnt activation induces ectopic *foxj1a* expression and ectopic cilia formation, possibly secondary to ectopic KV development. We revealed two distinct roles of Foxj1a in conferring Wnt-governed LR patterning. While Wnt controls cilia outgrowth via the canonical role of Foxj1a in ciliogenesis, it regulates *charon* expression via a novel non-ciliary function of Foxj1a.

## RESULTS

### Wnt activation promotes *foxj1a* transcription and induces ectopic *foxj1a* and ectopic cilia

**Fig. 1.**
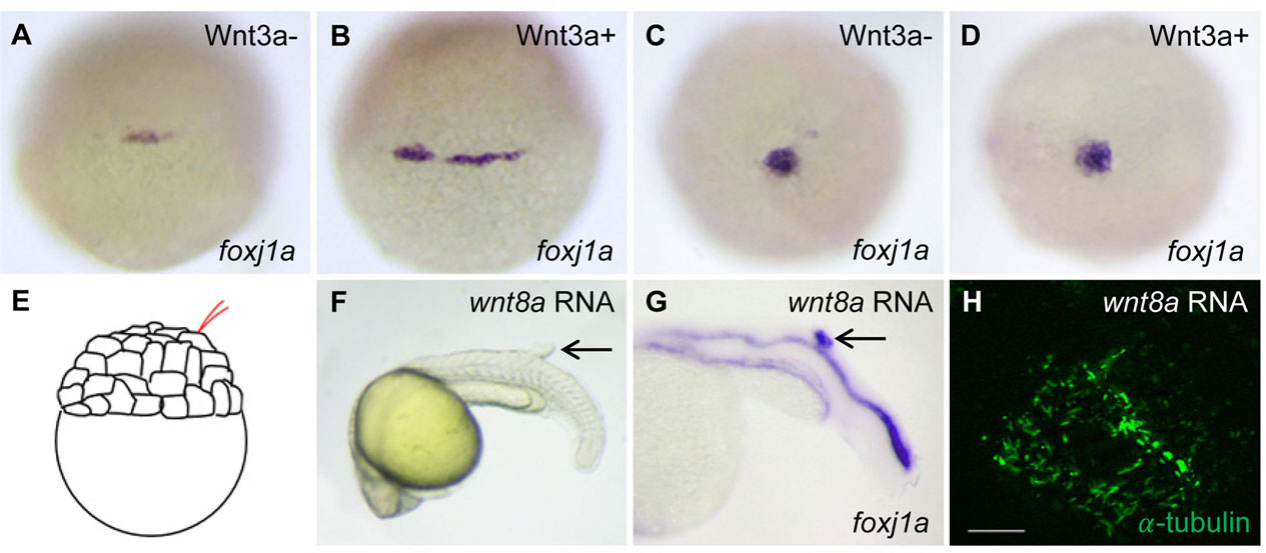
**The effect of Wnt activation on *foxj1a* expression and cilia formation.** (A-D) Wnt3a induction transiently enhances *foxj1a* levels in DFCs. *Tg(hsp:wnt3a-GFP)* embryos were heat activated at 30% epiboly for 30 min, and GFP+ (Wnt3a+) embryos were selected under fluorescence microscope. *foxj1a* transcript levels were examined at 1 h (A,B) and 4 h (C,D) after heat shock. Shown are dorsal views of embryos at 50% epiboly (A,B) and 95% epiboly (C,D). (E-H) Targeted overexpression of Wnt8a results in ectopic *foxj1a* expression and ectopic cilia formation. Schematic diagram shows a single cell injection at the 128-cell stage (E). Injection of *wnt8a* mRNA (100 pg) into a single cell at the 128-cell stage induced an ectopic protrusion (arrow in F), ectopic *foxj1a* expression (arrow in G, 8/8), and ectopic cilia-like structures as manifested by immunostaining using an antibody against acetylated α-tubulin (H). Whole-mount immunostaining was performed first, and then the protrusion was removed and imaged. Scale bar: 20 µm.

Given that Wnt/β-catenin signaling is required for *foxj1a* expression and ciliogenesis (Caron et al., 2012), we set out to test the effect of gain-of-Wnt-function. Our previous studies showed a transient activation of *foxj1a* in the zebrafish dorsal forerunner cells (DFCs) by inducible expression of β-catenin1, although steady-state expression of *foxj1a* was not altered by overexpression of Wnt3a, Wnt8a, and β-catenin1 (Caron et al., 2012). To validate the transient activation, we used an inducible transgenic *Tg(hsp:wnt3a-GFP)* strain. The *foxj1a* transcript level was increased at 1 h after Wnt3a induction (Fig. 1A,B), but returned to a level comparable to that of wild-type embryos at 4 h after heat shock (Fig. 1C,D). In contrast to causing transient upregulation of *foxj1a* in DFCs, Wnt3a and β-catenin1 activation was found to continuously increase *foxj1a* expression in the developing pronephros, another tissue in which Wnt is required for *foxj1a* expression (Fig. S1A-D; data not shown) (Caron et al., 2012).

To seek additional evidence to support a positive regulation of Wnt signaling on *foxj1a* transcription, we adopted a targeted injection strategy, i.e. injection of mRNAs into a single cell at the 128-cell stage, which allows important developmental pathways to be strongly activated without serious disruption of general development (Fig. 1E) (Agathon et al., 2003). Consistent with our previous report, a tailbud-like protrusion was induced in 20.3% (48/241) of *wnt8a* mRNA-injected embryos (Fig. 1F) (Lin et al., 2007). Importantly, all protrusions that were examined expressed ectopic *foxj1a* (Fig. 1G). In addition, they contained ectopic cilia-like structure as revealed by acetylated α-tubulin immunostaining (Fig. 1H). Together, these data suggest that Wnt activation induces *foxj1a* expression and ectopic cilia formation.

### Lef1 and Tcf7 cooperatively regulate *foxj1a* expression and ciliogenesis in KV via binding to the *foxj1a* regulatory sequence

**Fig. 2.**
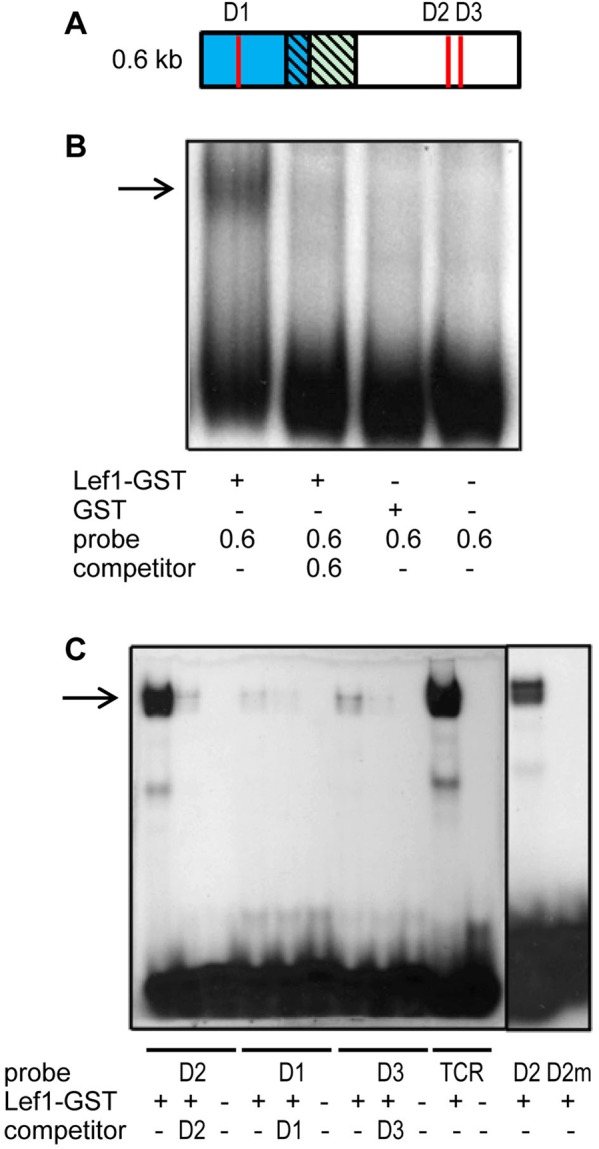
**Lef1 binds to the *foxj1a* regulatory sequence.** (A) Schematic diagram of a 0.6 kb *foxj1a* regulatory sequence. This sequence is located approximately −5.2 kb to −4.6 kb upstream of the ATG start codon. D1, D2, and D3 represent putative Lef1/Tcf binding sites. (B) Lef1 interacts with the *foxj1a* enhancer by electrophoretic mobility shift assay. Lef1-GST fusion protein (100 ng) or glutathione S-transferase (GST) alone (100 ng) was incubated with a ^32^P-labeled 0.6-kb *foxj1a* regulatory sequence in the presence or absence of 50-fold excess of cold probe. The resulting products were loaded onto a 4% acrylamide non-denaturing gel. (C) Lef1 predominantly binds to the D2 site. A Lef1-GST fusion protein (100 ng) was incubated with ^32^P-labeled oligonucleotides corresponding to D1, D2, D3, and mutated D2 (D2m) sites in the presence or absence of 100-fold excess of unlabeled oligonucleotide. The reaction mixtures were loaded into a 6% acrylamide non-denaturing gel. TCR indicates T-cell antigen receptor enhancer AAGTTTC motif, serving as a positive control for Lef1 binding.

To further elucidate the Wnt-Foxj1a signaling axis, we endeavored to define the transcription circuits that regulate *foxj1a* expression. Among five members of the Lef/Tcf family that have been identified in zebrafish (*lef1, tcf7, tcf7l1a, tcf7l1b,* and *tcf7l2*) (Dorsky et al., 1999; Kim et al., 2000; Young et al., 2002; Veien et al., 2005), *lef1* and *tcf7* transcripts were detected in DFCs (Fig. S2A) and near or within KV (Caron et al., 2012). We then performed a chromatin immunoprecipitation assay to test for a physical interaction between Lef1 and a 0.6-kb *foxj1a* regulatory sequence, which harbors three putative Lef/Tcf binding sites (D1, D2, and D3) and recapitulates the endogenous *foxj1a* expression pattern (Fig. 2A) (Caron et al., 2012). Lef1 coprecipitated with a distal ∼0.2-kb *foxj1a* fragment containing D1 site and a proximal ∼0.2-kb fragment containing D2 and D3 sites (Fig. S2B). To validate this association as direct protein-DNA interaction, electrophoretic mobility shift assay was performed. A Lef1-GST fusion protein, but not GST alone, bound efficiently to the 0.6-kb *foxj1a* fragment, and this binding could be specifically outcompeted with excess cold probe (Fig. 2B). Moreover, Lef1 bound predominantly to the D2 sequence but failed to interact with a mutated D2 oligonucleotide that disrupts the consensus Lef1 recognition sequence (Fig. 2C). Similarly, Tcf7 also bound predominantly to the D2 sequence (Fig. S2C). These biochemical studies suggest that Lef1 and Tcf7 directly bind to the same consensus site of the *foxj1a* promoter region.

**Fig. 3.**
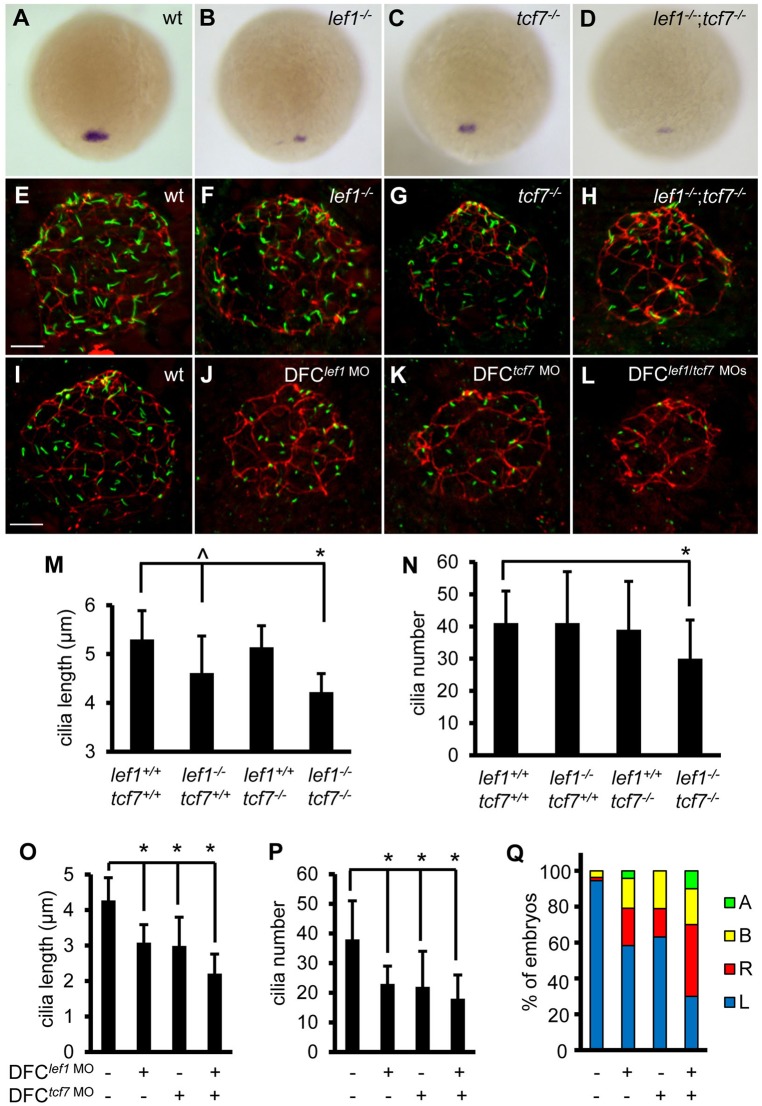
**Lef1 and Tcf7 cooperatively regulate *foxj1a* expression and ciliogenesis.** (A-D) Disruption of Lef1 or Tcf7 downregulates *foxj1a* expression in DFCs. *foxj1a* transcript levels were reduced in homozygous *lef1* (*lef1^−/−^*) (B) or *tcf7* (*tcf7^−/−^*) embryos (C) compared with wild-type siblings (A), and further decreased or abolished in double homozygous (*lef1^−/−^*;*tcf7^−/−^*) mutants (D). Shown are dorsal views of 95% epiboly staged embryos. Genotyping was performed on embryos that had been analyzed by *in situ* hybridization. (E-H) Disruption of Lef1 and Tcf7 affects ciliogenesis in KV. Embryos were fixed at the 12-somite stage, and the head regions were collected for genotyping. Then, immunostaining was performed on wild-type siblings and homozygous mutants. KV cilia were visualized by anti-acetylated α-tubulin staining (green) and apical-basal polarity of KV cells by anti-ZO-1 staining (red). (I-L) DFC-specific depletion of Lef1 or Tcf7 results in shorter and fewer cilia. *lef1* MO (2 ng) and/or *tcf7* MO (2 ng) were injected into the yolk cells at the 256-cell stage, and embryos were collected at the 10-somite stage for immunostaining. (M-P) Quantification of cilia length and number in mutants (M,N; 11–32 embryos were scored for each genotype) and morphants (O, P; 11–31 embryos were analyzed for each group). Data are represented as mean±s.d. (Q) DFC-specific depletion of Lef1 or Tcf7 randomizes left-sided *spaw* expression. Percentage of embryos with specific patterns of *spaw* expression was scored at the 21-somite stage. wt, wild type; A, absence; B, bilateral; L, left side; R, right side. ^*P*<0.05, * *P*<0.01 compared with controls. Scale bars: 20 µm in E-L.

Next, we conducted genetic studies to assess the role of Lef1 and Tcf7 in *foxj1a* expression in DFCs and ciliogenesis in KV by employing mutant *lef1^nl2/nl2^* and *tcf7^nkhg21cEt^^/nkhg21cEt^* zebrafish (Nagayoshi et al., 2008; McGraw et al., 2011). While a moderate reduction in *foxj1a* transcript level was observed in 23.9% (32/134) of offspring from a heterozygous *lef1^nl2/+^* incross or 20% (26/130) from a heterozygous *tcf7^nkhg21cEt^^/+^* incross, further reduction of *foxj1a* transcript levels was noted in 6.3% (21/331) of offspring from a double heterozygous *lef1^nl2/+^;tcf7^nkhg21cEt^^/+^* incross. Genotyping analysis confirmed that homozygous *lef1*, *tcf7*, and *lef1*;*tcf7* mutants have reduced *foxj1a* expression in DFCs (Fig. 3A-D). Homozygous *lef1* mutants had shorter KV cilia (4.61±0.76 μm vs 5.30±0.59 μm in wild-type siblings, *P*=0.012) but maintained normal cilia number (41±16 relative to 41±10, *P*=0.91) (Fig. 3F,M,N). Cilia in homozygous *tcf7* embryos were similar to those in wild type (length 4.86±0.44 μm, *P*=0.09; number 39±15, *P*=0.52) (Fig. 3G,M,N). Cilia in the double homozygous *lef1*;*tcf7* mutants were significantly shorter (4.22±0.38 μm, *P*=1.94×10^-5^) and fewer (30±12, *P*=0.009). Decrease in cilia number is less likely due to a reduction in DFCs because *sox17* expression was not altered (Fig. S3B).

We then asked whether Lef1 and Tcf7 regulate *foxj1a* expression KV cell-autonomously. DFC-targeted injection of *lef1* MO inhibited *foxj1a* transcript level (Fig. S3A) and significantly reduced cilia length (3.08±0.51 μm vs 4.37±0.64 μm in controls, *P*=6.54×10^-11^) and number (23±6 vs 39±10, *P*=5.21×10^-7^) (Fig. 3I,J,O,P). DFC*^tcf7^* ^MO^ embryos exhibited similar decreases in *foxj1a* expression (Fig. S3A) and ciliogenesis (length 2.99±0.81 μm, *P*=1.56×10^-6^; number 22±12, *P*=9.03×10^-4^). Further reduction in cilia length (2.21±0.55 μm) but not numbers (18±8) was observed in DFC*^lef1^* ^MO+*tcf7* MO^ embryos (Fig. 3I-L,O,P). Comparing with mutants, *lef1* or *tcf7* morphants had shorter and fewer cilia, even when normalized by KV size (data not shown). We attributed the more severe defects to a more complete disruption of Lef1 or Tcf7 function by MO injection. Indeed, the *lef1* MO targets a splice donor site at exon7/intron7, which is more upstream than the point mutation in the last exon of the *lef1^nl2^* allele (Ishitani et al., 2005; McGraw et al., 2011); the *tcf7*-ATG MO is expected to disrupt both maternal and zygotic mRNA translation, while maternal *tcf7* mRNA remains functional in the *tcf7* mutants (Nagayoshi et al., 2008). Consistent with KV cilia defects, LR patterning, as revealed by left-side-specific *spaw* expression in LPM, was cooperatively disrupted by knocking down Lef1 or Tcf7, or both [DFC*^lef1^* ^MO^ embryos: 58% left, 21% right, 17% bilateral, 4% absence (*n*=72); DFC*^tcf7^* ^MO^ embryos: 63% left, 16% right, 21% bilateral (*n*=57); DFC*^lef1^* ^MO+*tcf7* MO^ embryos: 30% left, 40% right, 20% bilateral, 10% absence (*n*=60)] (Fig. 3Q). Collectively, these genetic results suggest that Lef1 and Tcf7 cooperatively regulate *foxj1a* expression, KV ciliogenesis, and LR axis determination in a KV cell-autonomous manner.

To validate the role of Lef1 in mediating Wnt in ciliogenesis, we overexpressed Lef1 in embryos depleted of Fzd10, a proven receptor for Wnt/β-catenin signaling (Caron et al., 2012). Because Lef1 lacks transactivation activity, a constitutively active form of Lef1 (CALef1) was generated. Injection of *CAlef1* mRNA resulted in expanded expression of known Wnt targets, such as *chordin* and *ntla* (Fig. S4A-D), proving the functionality of the fusion protein (Lin et al., 2007; Martin and Kimelman, 2008). DFC-targeted injection of *CAlef1* mRNA rescued *foxj1a* levels, significantly restored cilia length (from 2.86±0.6 μm to 4.16±0.7 μm, *P*=1.02×10^-5^), and less significantly increased cilia number (from 22±8 to 30±12, *P*=0.02) in DFC*^fzd10^* ^MO^ embryos (Fig. S4E-G,I-K,M,N). CALef1 overexpression also restored left-sided *spaw* expression from 45% in DFC*^fzd10^* ^MO^ embryos to 72%, a level comparable to that of *CAlef1* mRNA injection alone (Fig. S4O). Consistent with other methods to activate Wnt signaling, injection of *CAlef1* mRNA alone into DFCs did not significantly affect steady state *foxj1a* expression and ciliogenesis (Fig. S4H,L). These results justified the use of CALef1overexpression for our further gain-of-Wnt-function studies.

### Targeted overexpression of Lef1 induces ectopic DFC lineage, KV-like structure, *foxj1a* expression, and cilia formation

**Fig. 4.**
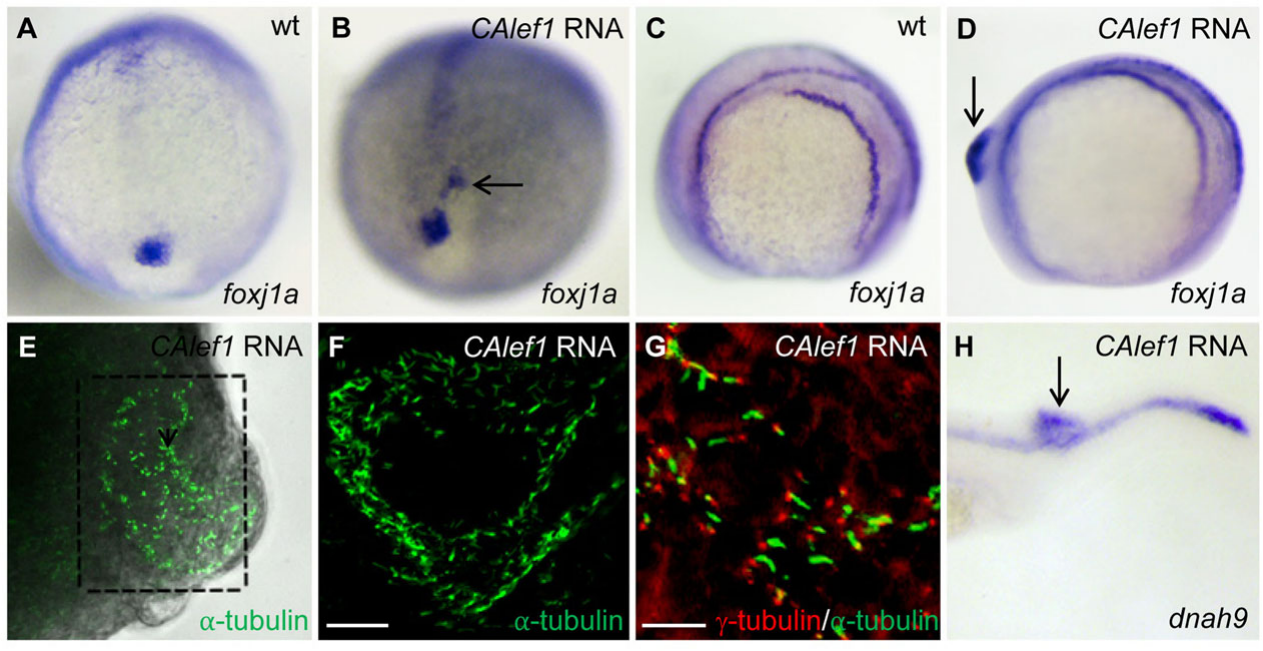
**Targeted overexpression of CALef1 induces ectopic *foxj1a* and ectopic Cilia.** (A, B) *CAlef1* mRNA (100 pg) was injected into 1-cell staged embryos. Ectopic *foxj1a* expression was observed (arrow in B). (C-H) *CAlef1* mRNA (100 pg) was injected into a single cell at the 128-cell stage of embryos. Embryos with an induced ectopic protrusion were collected at 10 to 14 somites (D-G) and 21 somites (H). The ectopic protrusion contained ectopic expression of *foxj1a* (arrow in D) and *dnah9* (arrow in H), and ectopic structures positive for anti-acetylated α-tubulin antibody staining (E, indicated by a dotted box, 5× magnification; amplified in F, 20× magnification). α-tubulin staining (green) was adjacent to γ-tubulin staining (red) (G). Shown are images taken from dissected and flattened protrusions (E,F) and JB-4 sections of the protrusions (G). Scale bars: 20 µm in F and 10 µm in G.

**Fig. 5.**
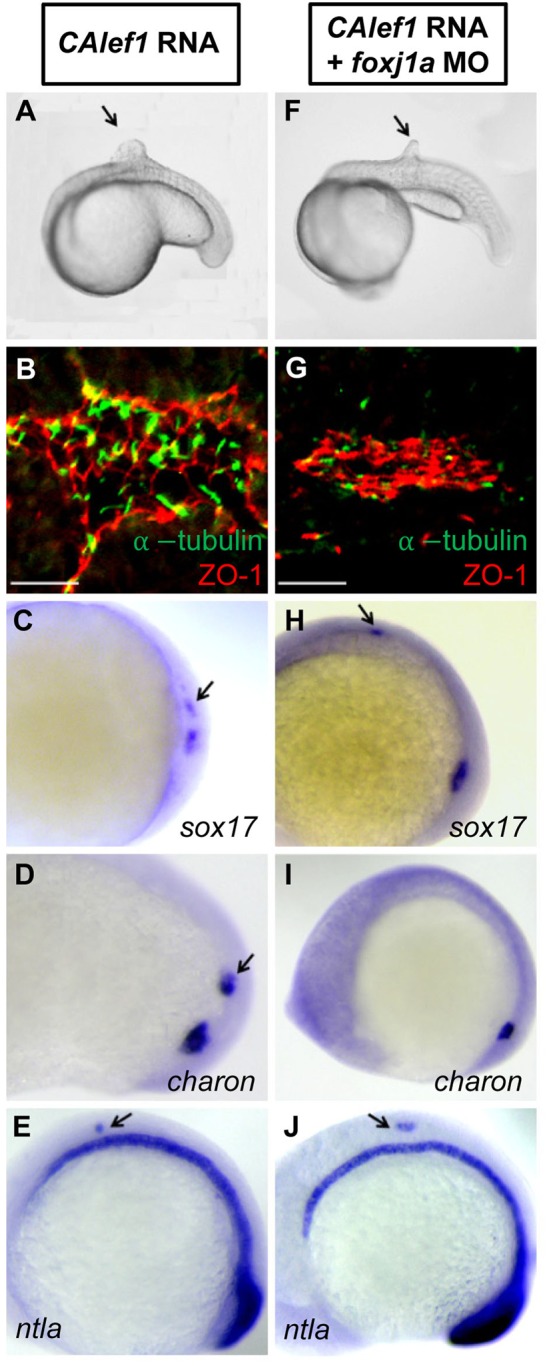
**Foxj1a mediates CALef1-induced ectopic cilia formation, not tissue formation.** (A-E) Injection of *CAlef1* mRNA (100 pg) into a single cell at the 128-cell stage resulted in formation of an ectopic protrusion (A), formation of ectopic cilia and ZO-1-positive cells (B), and ectopic expression of *sox17* (C), *charon* (D), and *ntla* (E). (F-J) Coinjection of *foxj1a* MO (150 pg) with *CAlef1* mRNA (100 pg) into a single cell at the 128-cell stage shortened ectopic cilia length (G) and abolished ectopic *charon* expression (I), but had no effect in the formation of an ectopic protrusion (F) or ectopic expression of ZO-1 (G), *sox17* (H), and *ntla* (J). Shown are embryo morphology at the 16- to 20-somite stage (A and F), *in situ* staining at 10 somites (C-E,H-J), and JB-4 sections of the protrusions after whole-mount double immunohistochemical labeling using antibodies against ZO-1 (red) and acetylated α-tubulin (green) (B and G). The arrow indicates ectopic protrusions (A, F) and ectopic gene expression (C-E,H,J). Scale bars: 20 µm.

Injection of *CAlef1* mRNA at the 1-cell stage resulted in ectopic *foxj1a* expression in a region near DFCs (18 of 146 injected embryos; Fig. 4B), while injection of *CAlef1* mRNA into a single cell at the 128-cell stage induced more profound tailbud-like protrusions in 25% (72/288) of injected embryos (Figs 4D and 5A). Compared with Wnt8a-induced ectopic structures, these protrusions are more visually identifiable, facilitating later experiments. Consistent with Wnt8a overexpression, CALef1-induced protrusions expressed ectopic *foxj1a* (Fig. 4D), and contained ectopic monocilia as assessed by acetylated α-tubulin antibody staining (Fig. 4E,F) and costaining with antibodies against basal body marker γ-tubulin (Fig. 4G) or another cilium marker, Arl13B (Fig. S5A). Transmission electron microscopy analysis further revealed tubular structure of a cilium (Fig. S5B). The ectopic cilia have an average length of 4.42±0.31 µm, which is similar to that of KV cilia and much longer than that of primary cilia (∼1 μm) (Yuan et al., 2012). In addition, genes exclusive to motile cilia, such as *dnah9* (dynein axonemal heavy polypeptide 9), *efhc1* (EF hand-containing protein 1), and *wdr78* (WD repeat domain 78) were detected in the protrusions (Fig. 4H; data not shown) (Hellman et al., 2010), suggesting that ectopic cilia induced by CALef1 are motile. Together, these data confirmed that Wnt activation is sufficient for *foxj1a* induction and ectopic cilia formation.

The Wnt/β-catenin pathway plays pleiotropic roles during embryonic development, including cell fate specification, cell type differentiation, stem cell maintenance, cilia regulation, and tissue morphogenesis (van Amerongen and Nusse, 2009; Caron et al., 2012; Clevers and Nusse, 2012; Zhang et al., 2012). The presence of monocilia similar to KV cilia in length prompted us to test the hypothesis that CALef1 is sufficient to induce the formation of KV-like cells in ectopic protrusions. Indeed, we detected the expression of *sox17*, a DFC lineage marker, and *charon*, a Nodal antagonist that is expressed only in the cells lining the KV in teleosts and is required for LR patterning, in CALef1-induced protrusions (Fig. 5C,D) (Alexander et al., 1999; Hashimoto et al., 2004). Additionally, we detected tight junction marker ZO-1 staining in the cilia-forming area of the protrusions (Fig. 5B), suggesting the presence of differentiated epithelial cells. Ectopic expression of *sox17*, *charon*, and ZO-1 was also observed after *wnt8a* mRNA injection (data not shown). In contrast, expression of markers for other motile cilia-containing tissues, such as *pax2a* and *cdh17* for pronephros and *nkx2.2b* and *ntn1b* for floor plate, was not observed in the protrusions (data not shown) (Norton et al., 2005; Wingert et al., 2007). Thus, targeted Wnt activation is capable of specifying progenitor cells to DFC lineage and differentiating them into *foxj1a*-expressing, cilia-forming, and *charon*-expressing KV-like cells.

### Foxj1a confers Wnt-governed cilia formation and *charon* expression

The robust CALef1-induced protrusion provides an experimental platform to dissect which aspects of Wnt functions are conferred by Foxj1a. Injection of *foxj1a* mRNA into a single cell at the 128-cell stage failed to induce ectopic protrusions, *sox17* expression, and *charon* expression despite its ability to elicit cilia formation (Fig. S6; data not shown) (Yu et al., 2008), suggesting that not all functions of Wnt/β-catenin signaling are conferred by Foxj1a. In embryos coinjected with *CAlef1* mRNA and *foxj1a* MO, knockdown of Foxj1a had no effect on the CALef1-induced ectopic protrusion (Fig. 5F), ectopic *sox17* expression (Fig. 5H), and ectopic ZO-1 expression (Fig. 5G). We also checked tailbud markers, such as *ntla,* that can be observed in Wnt-induced protrusions (Lin et al., 2007). Consistently, CALef1 activated ectopic *ntla* expression (Fig. 5E), which was not affected by Foxj1a knockdown (Fig. 5J). In contrast, we found that ectopic cilia were missing or shorter (1.37±0.26 µm, Fig. 5G) and ectopic *charon* expression was abolished on Foxj1a knockdown (Fig. 5I).

**Fig. 6.**
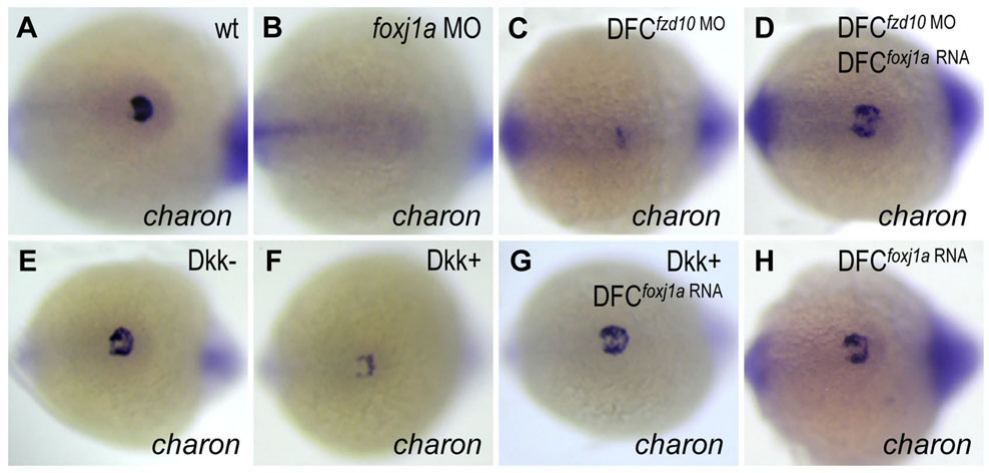
**Foxj1a confers Wnt-regulated *charon* expression in KV.** (A,B) Foxj1a is necessary for *charon* expression. Injection of *foxj1a* MO (2 ng) into 1-cell staged embryos led to reduction of *charon* levels (B, 18/20). (C-H) Foxj1a overexpression rescues *charon* levels in embryos with reduced Wnt signaling. *charon* transcript levels were downregulated by DFC-targeted injection of *fzd10* MO (4 ng) (C, 28/29) and by Dkk1 induction (F, 26/30), which were restored by injection of *foxj1a* mRNA (2 ng) (D, 23/28; G, 25/32). Injection of *foxj1a* mRNA alone did not significantly affect *charon* expression (H, 16/16). *Tg(hsp:dkk1-GFP)* embryos were heat shocked at 70% epiboly, and GFP+ (Dkk1+) embryos were selected under fluorescence microscope. Shown are dorsal views of tailbud region at 10 to 12 somites.

The abolished *charon* expression led us to speculate that Foxj1a might confer functions of the Wnt/β-catenin pathway in regulating *charon* expression in KV (Lin and Xu, 2009; Zhang et al., 2012). During embryogenesis, *charon* transcription is initiated at the 6-somite stage when *foxj1a* transcript levels in KV cells are already downregulated and barely detectable (Hashimoto et al., 2004; Caron et al., 2012). The expression profile also supports that Foxj1a might function upstream of *charon*. Consistently, injection of *foxj1a* MO at the 1-cell stage downregulated *charon* transcript levels in KV (Fig. 6B). To evaluate the role of Foxj1a in Wnt-*charon* regulation, we performed *foxj1a* rescue experiments. While injection of *foxj1a* mRNA alone did not significantly alter *charon* transcription (Fig. 6H), it partially rescued *charon* levels in DFC*^fzd10^* ^MO^ embryos (Fig. 6C,D) and Dkk1-expressing embryos (Fig. 6F,G). The epistatic analysis strongly suggests that Foxj1a mediates Wnt-regulated *charon* expression.

### The role of Foxj1a in *charon* expression is independent of its ciliogenic function

Members of Cerberus/Dan family Nodal antagonists, including *charon* in teleosts, are the first molecules exhibiting asymmetric expression around the node. The interplay between these Nodal inhibitors and Nodal has been shown to provide the signals that lead to the establishment of laterality (Hojo et al., 2007; Oki et al., 2009; Schneider et al., 2010; Schweickert et al., 2010; Kawasumi et al., 2011). While their asymmetric expression is thought to be generated by directional cilia-driven fluid flow and further enhanced by asymmetric posttranscriptional decay of mRNA, the transcription of *charon* seems not dependent on cilia motility (Gourronc et al., 2007; Nakamura et al., 2012). To clarify the relationship among Foxj1a, cilia motility, and *charon* transcription, we inspected the following mutants with defective motile cilia: *oval* (*ift88^tz288b/tz288b^*) whose KV cilia are shorter or missing and cilia beating is barely detectable, *lok* (*ccdc40 ^to237b/to237b^*) that have reduced cilia length and motility, and *ntla^b195/b195^* that harbor severely shorter or missing cilia in KV (Kramer-Zucker et al., 2005; Amack et al., 2007; Sullivan-Brown et al., 2008; Becker-Heck et al., 2011). *oval* and *lok* mutants exhibited normal *foxj1a* expression and, concordantly, normal *charon* transcript level (Fig. S7B,D,F,H). In contrast, *ntla* mutants exhibited loss of *foxj1a* and, concordantly, loss of *charon* (Fig. S7J,N). The lack of *foxj1a* and *charon* expression was not due to absence of DFCs because *sox17* was still expressed (Fig. S7L). Importantly, ectopic expression of Foxj1a rescued *charon* levels in *ntla* mutants (Fig. S7O). Taken together, these correlated data support that Foxj1a determines *charon* transcription while cilia beating initiates its asymmetric expression around KV.

**Fig. 7.**
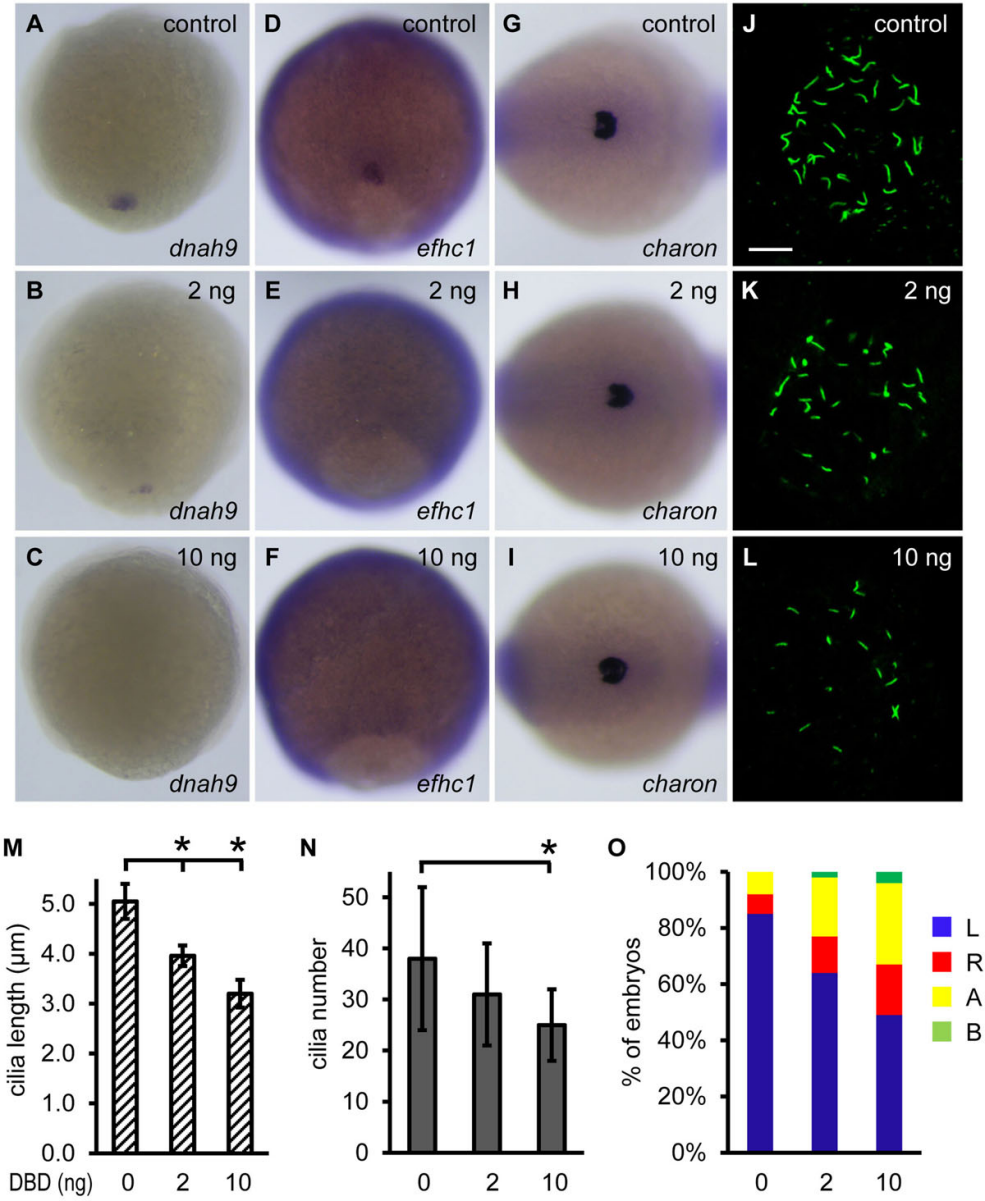
**Non-ciliary function of Foxj1a regulates *charon* expression.** (A-F) Overexpression of DBD-Foxj1a inhibits Foxj1a target gene expression. Injection of 2 ng of *DBD-Foxj1a* mRNA suppressed *dnah9* (B, 8/14) and *efhc1* (E, 6/14) transcript levels, and injection of 10 ng of mRNA nearly abolished *dnah9* (C, 18/21) and *efhc1* (F, 13/15) expression. Shown are dorsal views of 90% to 95% epiboly staged embryos. (G-I) Injection of *DBD-Foxj1a* mRNA did not affect *charon* expression at doses of either 2 ng (H) or 10 ng (I). Shown are dorsal views of tailbud region at 10 to 12 somites. (J-L) Overexpression of DBD-Foxj1a results in defective ciliogenesis. Shorter (K,L) and fewer (L) KV cilia were visualized at the 10-somite stage by anti-acetylated α-tubulin staining. Scale bar: 20 µm. (M,N) Quantification of cilia length and number. 9 to 11 embryos were analyzed for each group. Data are represented as mean±s.d. *P<0.01 compared with controls. (O) Overexpression of DBD-Foxj1 randomizes left-sided *spaw* expression. Percentage of embryos with specific patterns of *spaw* expression was scored at the 21-somite stage. DBD, DNA-binding domain; A, absence; B, bilateral; L, left side; R, right side.

Foxj1 has been considered a master transcription factor for motile cilia biosynthesis; it also induces genes that are required for ciliary differentiation and cilia beating (Chen et al., 1998; Stubbs et al., 2008; Yu et al., 2008; Choksi et al., 2014). Judging from the time course of *foxj1a* and *charon* expression in DFCs and KV, *charon* is less likely a direct transcriptional target of Foxj1a. To test this possibility and to discern roles of Foxj1a in ciliary gene induction and *charon* expression, we overexpressed DNA-binding domain of Foxj1a (DBD-Foxj1a) (Hackett et al., 1995). DBD-Foxj1a is expected to compete with endogenous Foxj1a for binding to the promoter region of Foxj1a-inducible genes, thus acting as a dominant negative form of Foxj1a via abolishing its transcriptional activity. Meanwhile, endogenous Foxj1a remains intact and presumably still executes other functions independent of its transcriptional activity. We found that injection of 2 ng of *DBD-Foxj1a* mRNA resulted in downregulation of *dnah9* and *efhc1*, two Foxj1a target genes that are structural and functional components of the ciliary apparatus (Fig. 7B,E) (Stubbs et al., 2008; Yu et al., 2008; Choksi et al., 2014). As a consequence, KV cilia were significantly shorter (3.96±0.21 μm compared with 5.05±0.35 μm in wild-type embryos, *P*=8.37×10^-4^) and marginally fewer (31±10 vs 38±14, *P*=0.20) (Fig. 7K,M,N), and left-sided *spaw* expression was randomized (64% left, 13% right, 21% bilateral, 2% absence [*n*=128]) (Fig. 7O). Injection of a higher amount of *DBD-Foxj1a* mRNA (10 ng) abolished *dnah9* and *efhc1*expression, further reduced cilia length (3.20±0.28 μm, *P*=2.88×10^-7^) and number (25±7, *P*=4.97×10^-3^), and enhanced abnormal *spaw* expression [49% left, 18% right, 29% bilateral, 4% absence (*n*=108)] (Fig. 7C,F,L-O). In contrast, *charon* expression levels remained unaffected by DBD-Foxj1a overexpression (Fig. 7H,I), indicating that Foxj1a-regulated *charon* expression is independent of the canonical role of Foxj1a as a ciliogenic transcription factor.

## DISCUSSION

**Fig. 8.**
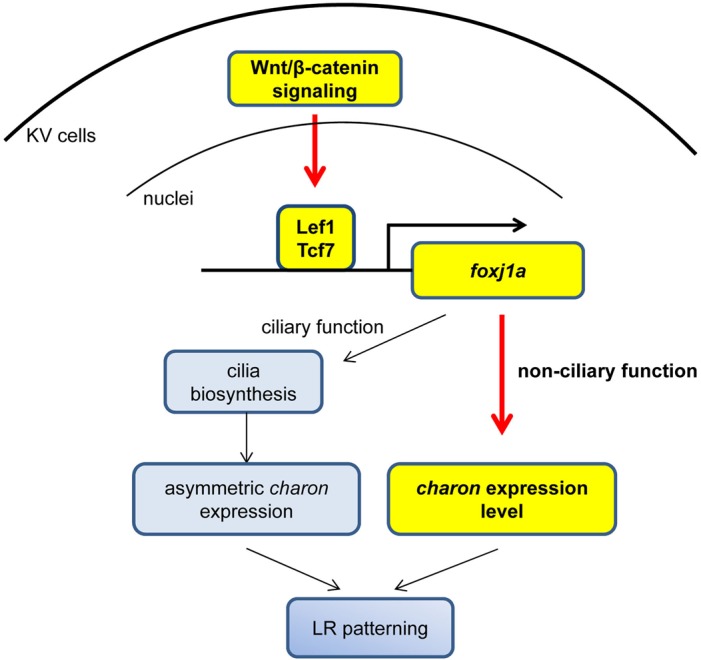
**Summary of functions of Wnt-Foxj1a signaling in LR pattern formation.** Wnt/β-catenin signaling regulates Foxj1a expression in KV via Lef1 and Tcf7 binding to the *foxj1a* regulatory sequence. The Wnt-Foxj1a axis appears to regulate LR asymmetry via at least two mechanisms: the first involves cilia outgrowth that is dependent on the canonical function of Foxj1a in cilia formation, and the second involves expression of *charon* that requires non-ciliary function of Foxj1a. Yellow highlighting indicates novel discoveries of the study.

As described in this article, we revealed the transcription circuit of *foxj1a* regulation and validated our previous finding that Foxj1a is a direct transcriptional target of Wnt (Caron et al., 2012). We further uncovered two critical roles that the Wnt-Foxj1a axis plays during LR pattern formation. First, Wnt regulates cilia growth via a ciliary function of Foxj1a. This is a known function of Foxj1a that is dependent on its ciliogenic function. Second, Wnt determines *charon* transcription via a non-ciliary function of Foxj1a. This is a novel function of Foxj1a that is independent of its canonical role as a ciliogenic transcription factor. Because cilia defects will affect directional nodal flow and subsequent asymmetric expression of *charon*, we propose a model that ciliary and non-ciliary functions of Wnt-Foxj1a signaling converge at *charon* to regulate LR axis determination (Fig. 8). Our novel discovery of non-ciliary function of Foxj1a underlying Wnt-implicated laterality defect adds new knowledge to the field of LR asymmetry and might be applicable to other signaling pathways that regulate Foxj1a expression.

### Molecular nature of Wnt/β-catenin signaling in *foxj1a* activation

We presented both biochemical and genetic evidence to suggest that Lef1 and Tcf7 are transcription factors that cooperatively confer Wnt-regulated *foxj1a* transcription during KV development. Unlike severe disruption by inhibition of Wnt signaling (Lin and Xu, 2009; Caron et al., 2012), *lef1* and/or *tcf7* mutants exhibit moderate defects in *foxj1a* expression, ciliogenesis, and presumably, LR asymmetry. This phenomenon is likely attributable to maternal expression of Lef1 and/or Tcf7, which compensates for loss of zygotic Lef1 and/or Tcf7 in the mutants (Dorsky et al., 1999; Veien et al., 2005). Precedent for this scenario comes from the observation that cilia can still form in zygotic *oval* mutants but are absent in maternal-zygotic *oval* mutants during early embryogenesis (Huang and Schier, 2009). Implication of other Tcfs in KV ciliogenesis is less likely because we were unable to detect expression of other *tcfs* in DFCs or KV.

Besides Lef1 and Tcf7, several observations prompted us to propose that Wnt needs cofactors to fulfill its function on *foxj1a* expression. First, Wnt signaling has a broader functional domain than *foxj1a*, which exhibits a more tissue-restricted expression pattern (Aamar and Dawid, 2008; Yu et al., 2008). Second, Wnt activation enhances *foxj1a* levels transiently in DFCs but continuously in the developing pronephros. It is possible that a negative feedback mechanism is utilized in DFCs while a positive feed-forward mechanism is engaged in the pronephros. Third, Wnt activation induces ectopic *foxj1a* expression in a small proportion of cells, which might be fate-specified to express certain transcription cofactors. In support of our cofactor hypothesis, it has been reported that members of the Lef/Tcf family bind to target DNA weakly and with moderate specificity, which demands cooperative interactions with other factors to achieve tight and specific control of target gene regulation. For example, SMADs and members of the homeodomain family (Pitx2, Cdx-1, Nrarp) are engaged in cooperative interactions with Lef/Tcfs to regulate transcription of target genes (Labbe et al., 2000; Nishita et al., 2000; Beland et al., 2004; Ishitani et al., 2005; Vadlamudi et al., 2005). Identification of Lef1 and Tcf7 laid the foundation for our future search for Wnt cofactors in controlling *foxj1a* expression in KV, which will enable us to gain further insight into the molecular nature of this novel function of Wnt.

### Ciliary function of Foxj1a in mediating Wnt signaling

Overexpression of Foxj1 was shown to induce node-like monocilia in *Xenopus* and zebrafish by activating a large number of genes that encode components unique to motile cilia, including dynein arms, central pair, and radial spokes (Stubbs et al., 2008; Yu et al., 2008). Consistently, our data indicate that Foxj1a is responsible for cilia length elongation and cilia component expression. By employing a targeted Wnt activation system, we showed that Wnt is sufficient to induce a distinctive ectopic tissue protrusion, which can be used to conduct gain-of-Wnt-function analysis. Ectopic expression of *foxj1a* was detected, and knocking down Foxj1a ablated the *de novo* cilia synthesis, underscoring the ciliary function of Wnt-Foxj1a signaling. This platform overcomes the shortcoming of early embryonic lethality resulting from global manipulation of important developmental pathways, and is ideal for future studies to identify pathways or molecules that are involved in Foxj1 expression and cilia synthesis. In addition to cilia growth, this platform is also useful for testing candidate genes that are implicated in cilia assembly. Given that the ectopic protrusion is morphologically distinguishable, *de novo* synthesized cilia can be identified for structural analysis via transmission electron microscopy.

Our past and present studies placed Wnt/β-catenin signaling upstream of ciliogenesis via direct transcriptional regulation of *foxj1a* (Caron et al., 2012). In contrast, functions of cilia upstream of Wnt signaling are contradictorily reported. Some studies suggest that cilia exert an inhibitory role on the Wnt/β-catenin pathway (Gerdes et al., 2007; Corbit et al., 2008) and are required for switching between the Wnt/β-catenin and Wnt/PCP pathways (Simons et al., 2005). However, other investigators argue against a function of cilia in regulating Wnt signaling (Huang and Schier, 2009; Ocbina et al., 2009). Of note, we observed tissue-specific *foxj1a* activation by Wnt, as evidenced by transient and persistent increase in DFCs and the developing pronephros, respectively. It remains to be determined whether *foxj1a* levels are differentially regulated by a feedback or a feed-forward mechanism to which cilia-Wnt regulation might contribute.

### Non-ciliary function of Foxj1a in transducing Wnt signaling

In addition to the canonical role of Foxj1a in ciliogenesis, our data reveal a novel function of Foxj1a in KV development, i.e. it confers Wnt-regulated *charon* expression. Unlike its function as a ciliogenic transcription factor, this novel function of Foxj1a is likely independent of its DNA-binding ability. Consistent with this non-ciliary function of Foxj1a, we and others found that cilia and cilia motility are not required for overall *charon* expression (Gourronc et al., 2007). In fact, non-ciliary functions of Foxj1 have also been suggested in other tissues, including the differentiation of radial glia into ependymal cells and a subset of astrocytes in the postnatal brain and specification of cellular lineage in the forebrain (Jacquet et al., 2009, 2011). On the basis of sequential expression of *foxj1a* and *charon* during KV development, Foxj1a might not directly regulate *charon* transcription. Instead, we favor the hypothesis that Foxj1a might affect *charon* expression through regulating KV cell differentiation.

Non-ciliary function of the Wnt-Foxj1 axis in asymmetry remains to be investigated in mammals. The LR axis is randomized in *Wnt3a* and *Foxj1* null mice, but cilia at the embryonic node are still present (Chen et al., 1998; Zhang et al., 2004; Nakaya et al., 2005). The presence of nodal cilia might be explained by the Rfx family of X-box-binding transcription factors, which act redundantly with Foxj1 to induce motile ciliogenesis in the neural tube and, presumably, in the mouse node but not in the zebrafish KV (Bonnafe et al., 2004; Yu et al., 2008; Cruz et al., 2010; Bisgrove et al., 2012). It would be informative to determine in the future whether *Cerl2*, whose deficiency results in a wide range of laterality defects, is downregulated in *Wnt3a* and *Foxj1* null mice (Marques et al., 2004). Strengthening the Wnt-*Cerl2* relationship, recent studies suggested that *Wnt3* is asymmetrically expressed in the crown cells of the node and enhances asymmetric decay of *Cerl2* mRNA (Nakamura et al., 2012; Kitajima et al., 2013). Nonetheless, these mechanistic studies will help us to better interpret related human diseases such as congenital heart diseases associated with LR defects.

## MATERIALS AND METHODS

### Zebrafish strains

Zebrafish were maintained in accordance with the policies of the Mayo Clinic Institutional Animal Care and Use Committee. Wild-type (TL), heterozygous *lef1^nl2/+^* and *tcf7^nkhg21cEt^^/+^* lines, and transgenic *Tg(hsp:dkk1-GFP)* and *Tg(hsp:wnt3a-GFP)* strains were used for this work (Nagayoshi et al., 2008; Hoage et al., 2011; McGraw et al., 2011; Caron et al., 2012). Genotyping of the *lef1^nl2/nl2^* and *tcf7^nkhg21cEt^^/nkhg21cEt^* embryos was performed according to protocols from Alexei V. Nechiporuk's laboratory (Oregon Health & Science University, Portland, Oregon, USA) and Koichi Kawakami's laboratory (National Institute of Genetics, Mishima, Shizuoka, Japan), respectively.

### Morpholino injections

Antisense morpholino oligonucleotides (MOs) (purchased from Gene Tools) targeting splice donor sites of *lef1* (*lef1* MO) and the translation initiation site of *tcf7* (*tcf7* MO), *fzd10* (*fzd10* MO), and *foxj1a* (*foxj1a* MO) have been described previously (Ishitani et al., 2005; Nagayoshi et al., 2008; Stubbs et al., 2008; Caron et al., 2012). To target MOs specifically to DFCs, MOs were injected into the yolk cell at the 256-cell stage (Amack and Yost, 2004).

### Cloning and RNA injections

Full-length zebrafish *lef1* cDNA was amplified by an Expand High Fidelity PCR System (Roche Life Science) using 24-h-post-fertilization cDNA as a template. CALef1 was generated by fusing the carboxyl-terminal domain of zebrafish β-catenin1 (694–780 aa) to the N-terminus of zebrafish full-length Lef1 (Hsu et al., 1998; Aoki et al., 1999; Galceran et al., 2001). DBA-Foxj1a (136–240 aa) was amplified from the full-length zebrafish *foxj1a* cDNA (Hackett et al., 1995). The resulting cDNA fragments were cloned into pCS2+ plasmids. Capped mRNAs for *lef1*, *CAlef1*, *DBD-Foxj1a*, *wnt8a*, *foxj1a*, and *gfp* were synthesized from their corresponding pCS2+ plasmids using the mMESSAGE mMACHINE SP6 Transcription Kit (Ambion) (Lin et al., 2007; Caron et al., 2012).

To perform targeted overexpression, mRNAs (100 pg), alone or together with MOs (150 pg), were injected into single cells at the animal pole of 128-cell staged embryos. *gfp* mRNA (100 pg) was coinjected to serve as a marker (Agathon et al., 2003; Lin et al., 2007). Embryos that harbored localized green fluorescence protein (GFP)-fluorescence signals or contained ectopic protrusions were collected at 10 to 21 somites for further analysis.

### *In situ* hybridization and immunofluorescence

Single-color, whole-mount *in situ* hybridization was performed as previously described (Xu et al., 2002). Cilia, basal body, and apical-basal polarization of KV cells were visualized using antibodies against acetylated α-tubulin (Sigma-Aldrich), γ-tubulin (Sigma-Aldrich), and the tight junction protein ZO-1 (Invitrogen) as previously described (Lin and Xu, 2009). Cilia length and number were measured using AxioVision software (Zeiss) and analyzed by the Student *t*-test. For analysis of the ectopic protrusion, immunostaining was performed in whole-mount embryos, and then the ectopic protrusion was dissected and mounted in VECTASHIELD (Vector Laboratories), or the embryos were embedded in JB-4 plastic resin (Polysciences) and sectioned at 4-μm sections (Sullivan-Brown et al., 2011).

### Electrophoretic mobility shift assay

Electrophoretic mobility shift assay was performed as previously described (Hellman and Fried, 2007). Full-length zebrafish *lef1* cDNA was cloned into a pGEX4T-1 plasmid. Lef1-GST fusion protein was expressed in *Escherichia coli* and purified using Glutathione-Sepharose 4B (GE Healthcare). Briefly, Lef1-GST fusion protein was incubated with a ^32^P-labeled 0.6-kilobase (kb) regulatory sequence of *foxj1a* in the presence or absence of excess cold probe or with ^32^P-labeled oligonucleotides corresponding to putative Lef/Tcf binding sites (Caron et al., 2012). The sequences for the oligonucleotide probes are GGGACTCTGTTTACAGGGGG (D1), TATTTATCCTTTGTTTAGAT (D2), TATTTATCCATAGTTTAGAT (D2m, mutated D2), and TACACCCACAGAGACATTTG (D3).
